# Psychosocial characteristics among older adult clients in outpatient care

**DOI:** 10.3389/fpsyt.2025.1640791

**Published:** 2026-01-23

**Authors:** Lisa Winter, Stella Becher-Urbaniak, Manuel Fürholzer, David Seistock, Dimitrios Külzer, Jan Aden

**Affiliations:** 1 Psychotherapeutic Outpatient Clinic for Adults, Sigmund Freud Private University Vienna, Vienna, Austria; 2 Institute for Statistics, Faculty of Psychology, Sigmund Freud Private University Vienna, Vienna, Austria

**Keywords:** psychotherapy, age, psychopathology, lifespan-development, gerontopsychology

## Abstract

**Introduction:**

This study investigates main psychosocial characteristics of older adults (age ≥60) in an outpatient psychotherapeutic context and to derive implications for tailored psychotherapeutic services by assessing the socio-demographic factors of this clientele, thereby deepening mental health professionals’ understanding of psychotherapy for this age group.

**Method:**

The processed data were acquired during the standard initial registration protocol at the psychotherapeutic outpatient clinic for adults of the Sigmund Freud Private University Vienna (SFU). An analysis of psychosocial and clinical characteristics was conducted for n = 166 older adult clients. To operationalise the psychopathological characteristics, the instrument Clinical Outcomes in Routine Evaluation – Outcome Measure (CORE-OM) was used. Thus, the results were obtained using one-way ANOVAs, χ^2^ analyses, and descriptive statistics.

**Results:**

Older adult clients differ mainly compared with the other age groups in the following characteristics: lower educational levels [19.3%; χ^2^(n = 166, 10) = 305.04, c = .24, c_corr_ = .28, p <.001], higher expression rates of somatic diseases [51.2%; χ^2^(n = 166, 2) = 24.21, c = .07, c_corr_ = .09, p <.001], need for psychopharmacological treatment [43.3%; χ^2^(n = 166, 2) = 171.19, c = .18, c_corr_ = .24, p <.001], and lower level of alcohol and drug abuse [86.7%; χ^2^(n = 166, 2) = 195.08, c = .19, c_corr_ = .26, p <.001]. Moreover, older adult clients indicate significantly lower well-being scores than other age groups [F(2, 5,042) = 8.18, p <.001, η^2^ = .003]. Additionally, older adults tend to terminate their therapeutic process prematurely (20.8%) but do not differ in effect systematically (p >.05).

**Discussion:**

This study demonstrates that older adult clients are similar to the other age groups in many psychosocial aspects but are additionally confronted with specific age-related challenges. Psychosocial care institutions should take these challenges into account.

## 1 Introduction

### 1.1 Peculiarities of psychotherapeutic care for older adult populations

As the world continues to experience a drastic demographic change regarding longevity, the population share of individuals aged 60 and above is constantly increasing. As reported by the United Nations Population Division, the number of persons aged 65 and above is expected to exceed the number of children aged 18 and below by 2080. Although this change is considered to be a global phenomenon, its effects are the most visible in Europe, North America, Australia, and New Zealand, calling for the need to develop and strengthen healthcare and social protection systems and support national policies to create inclusive societies for all ages (1). While this proposition is undoubtedly a noble goal, there are currently heated debates on how this can be best achieved without endangering existing social security structures, ranging from raising retirement ages to additional benefits for parents. Most solutions proposed come with an inherent additional burden placed on younger generations and are therefore widely unpopular throughout most of society.

However, another approach to addressing this issue is to train existing social care institutions and personnel to more effectively deal with the challenges that arise from ageing populations. The focus of such interventions is usually placed on physical health, as these issues are often clearly visible, can therefore be more easily assessed and addressed, and are generally more present in older populations. However, more recent research points to the bidirectionality of physical health and mental health, especially when it comes to physical activity (2), highlighting the necessity of positive affect for engagement in healthy behaviour. Therefore, neglecting mental health needs may lead to less physical activity and potentially worse physical health in turn.

This study aims to investigate the main psychosocial characteristics of older clients (age ≥60) in a psychotherapeutic outpatient context and to derive implications for tailored psychotherapeutic services via assessing socio-demographic factors of this clientele.

### 1.2 Using sociodemographic characteristics to inform clinical decision making

Although insights and particular conclusions vary across studies, especially when considering different populations, sociodemographic factors have long been widely acknowledged as substantial predictors for identifying at-risk populations and the efficiency and efficacy of mental health interventions (3–7). They are therefore reliable parameters to consider for clinical decision making and policy planning.

Given that the authors of this study are associated with a psychotherapeutic outpatient clinic in Vienna, keeping records of a wide range of sociodemographic markers to comply with documentation requirements, the authors recognise this opportunity to derive valuable insights from the data collected this way.

### 1.3 Benefits of introducing specialised care for older adult clients

As stated above, the effects of sociodemographic factors on mental health and health in general can be observed across studies, but implications may vary depending on the surveyed populations. The focus of this study on older adult clients bears significance to point out that past research has successfully identified social vulnerabilities such as frailty, functional decline and dependence, mobility, institutionalisation, behaviour (e.g., conforming with norms), and the effects of cognitive decline and dementia on social interactions (8). Laidlaw and Pachana (9) concluded that specialised psychotherapeutic approaches to effectively tackle the challenges confronted by older populations may not only be warranted but also necessary to deliver effective treatment, as psychotherapists’ preconceptions about ageing may negatively impact treatment outcomes.

## 2 Research questions

As stated in the introduction, untangling psychosocial burdens of age-related changes across life spans to explore specific challenges and adjustment processes in later adulthood helps psychotherapists to develop awareness and tailored support for clients.

Hence, the following research questions were derived for this purpose:

- Which specific sociodemographic characteristics and aspects of (bio-)psychosocial burdens define elder clients of the psychotherapy outpatient clinic for adults of the Sigmund Freud Private University Vienna?
- Do older clients differ in their (bio-)psychosocial stress profiles from clients in younger age groups?

The aim of this paper was to answer these formulated research questions, discuss their results as well, and appeal for further research in this field.

## 3 Materials and methods

Data were obtained naturalistically during the regular registration process at the psychotherapeutic outpatient clinic for adults of the Sigmund Freud Private University Vienna (SFU). After the subjects provided consent, sociodemographic, medical, and psychometric data were collected to screen clients for the severity of symptoms, optimal client allocation, and general research purposes. As this study focused on characteristics of geriatric clients, clients aged 60 and above were mainly enrolled. This project revealed an initial overview of the collected clinical gerontopsychological information (as of August 2024) at the outpatient clinic of the SFU.

### 3.1 Sampling

The accumulated data encompass a sample of n= 5050 individuals, which were filtered to access the gerontopsychological sample (clients age of 60 or above), which initially included n=166 persons who age, between 61 to 88 years. Socio-demographic characteristics of this sample are displayed in **Table 1**. All clients gave their consent to the anonymized use of the recording data for research purposes. The psychotherapeutic services offered are low-threshold and are intended for individuals who are still capable of making decisions and acting on their own behalf. The procedures comply with ethical standards for data protection and the protection of vulnerable population groups.

**Table 1:** Sociodemographic characteristics of the collected sample.

Dimensions	Mean	Standard deviation (SD)	-
Age	67.8	8.77	
Dimensions	Expression	Frequency	Percentage
Sex	Male	57	34.3%
	Female	109	65.7%
Educational level	No school degree	6	3.6%
	Compulsory school	32	19.3%
	Apprenticeship	38	22.9%
	A-levels	22	13.3%
	Academic degree	42	25.3%
	No data	26	15.7%
Working status	Unemployed	33	19.9%
	Retirement	75	45.2%
	Invalidity retirement	12	7.2%
	In training	2	1.2%
	Self-employed	6	3.6%
	Marginal pay	3	1.8%
	Half-time employment	7	4.2%
	Full-time employment	15	9%
	No data	13	7.8%
Relationship-status	Single	20	12%
	Unmarried with partner	9	5.4%
	Married	32	19.3%
	Divorced, no partner	32	19.3%
	Divorced, with partner	7	4.2%
	Widowed, no partner	17	10.2%
	Widowed, with partner	2	1.2%
	Separated	5	3%
	No data	42	25.3%
Parent	Yes	105	63.3%
	No	61	36.7%
Number of children	One child	37	22.3%
	Two children	45	27.1%
	Three children	16	9.6%
	Four children	6	3.6%
	Six children	1	0.6%
Citizenship	Austrian	128	77.1%
	Non-Austrian	38	22.9%
Birth country	Austrian	100	60.2%
	Non-Austrian	66	59.8%
Native language	German	125	75.3%
	Non-German	41	24.7%

### 3.2 Procedure and instruments

Regular registration procedure at the outpatient clinic requires clients to complete basic data, history of symptoms, and psychometric questionnaires. The data collection instruments for basic data and symptom history are proprietary, whereas the psychometric instruments are the “Clinical Outcomes in Routine Evaluation – Outcome Measure” (CORE-OM) by Sproll (10) and the short version of the World Health Organization Quality of Life questionnaire (11). For the present study, we were exclusively evaluating psychometric data acquired using the CORE-OM. The employed version is the German translation of the British original by C. Evans et al. (12).

The CORE-OM is a 34-item clinical questionnaire that assesses the subdimensions “well-being” (α = .77), “functioning” (α = .86), “risk (to self or others)” (α = .79), and “problems/symptoms” (α = .9), providing a total mean score of all subdimensions as well as a mean score for every subdimension separately. The questionnaire provides a Likert scale with a single-choice five-option response system (from “strongly disagree” to “strongly agree”). A higher score indicates higher pathological levels.

Moreover, through the clinical nature of the instrument, C. Evans et al. (13) defined scores that result in the subdimensions and total score that can be evaluated as clinically relevant. For this, the authors defined that scores above 1 (when calculating means) or higher than 10 (when calculating sums) characterise clinically significant psychopathological expressions, indicating a need for psychotherapeutic treatment.

### 3.3 Statistical analysis

Addressing the first research question required descriptive statistical analysis to be used for the evaluation of frequencies, percentages, and dispersion measures. This procedure provided us with the means to generate holistic geriatric psychopathological profiles for clients who have registered at the psychotherapeutic outpatient clinic for adults of the SFU.

Regarding the second research question, we firstly divided our sample into three age groups according to the categorisation provided by Newman and Newman (14) (early adulthood, 18–34 = 1; middle adulthood, 35–59 = 2; and late adulthood, ≥60 = 3). Secondly, based on this allocation, we calculated ANOVAs to compare the three age groups regarding CORE-OM scores. We also checked the statistical requirements, like the normal distribution or the homogeneity of variances. When data did not fulfil the presented requirements, we conducted alternative tests, like the Brown–Forsythe test or the Kruskal–Wallis H test. We generated effect sizes using (15) η^2^ effect size (small = .01, moderate = .06, and large = .14). Lastly, we introduced *post-hoc* analyses with pairwise tests (Bonferroni).

To compare the three categories (age groups) described above, χ^2^ analyses were used to further describe the diverse psychopathological profiles. As part of Pearson’s χ^2^ analyses, the following parameters were calculated: the contingency coefficient (c) and its corrected version (c<sub>corr</sub>), as well as standardised residuals (z) concerning sample-related frequency peculiarities, which indicate expression from higher (z = +1.96) to lower values (z = −1.96) than initially mathematically expected.

## 4 Results

In this chapter, the formulated hypotheses shall be statistically explored through inference, and the depicted data will be descriptively portrayed. Henceforth, the generated client information will be assessed in dependence on diverse sociodemographic and environmental conditions through computing frequencies, percentual distributions, and dispersion measures.

### 4.1 Results of the first research question: descriptive psychosocial profiles

A descriptive analysis of different domains was highlighted by this project. Thus, the following three facets were taken into consideration to holistically define the gerontopsychological profiles of the evaluated clients: the medical history, the therapeutic procedure, and the psychopathological load of the clients.

Beginning with the intra-individual need for medical attendance, we were able to measure (**Table 2**) that 43 (25.9%) of the 166 clients evaluated were hospitalised due to psychological issues. Furthermore, 75 individuals, who represent 45.2% of the sample, utilised psychological treatment at least once. While the majority (61.4%) did not indicate suffering from any physical illness, 64 of the 166 individuals questioned reported suffering from at least one somatic disease. These include dermatological (35.9%), gastrointestinal (50%), coronary (51.6%), pulmonary (48.4%), ocular (45.3%), or otolaryngologic (29.6%) illnesses. The most common somatic pathologies reported concern the musculoskeletal system, where 43 of the 64 somatically ill clients (67.2%) showed clinically significant deviances. Additionally, 72 (43.4%) of the clients included in the data set disclosed taking psycho-pharmaceuticals for therapeutic purposes. Across these cases, the medications prescribed are as follows: 53 (73.6%) individuals took antidepressants, 19 (26.3%) individuals were prescribed antipsychotics, and 13 (18.1%) clients were using anxiolytics (18.1%). Moreover, hypnotics (8.3%), mood stabilisers (12.5%), and psychostimulants (1.3%) were therapeutically administered as well.

**Table 2:** Sociodemographic characteristics of the medical history.

Dimensions	Expression	Frequency	Percentage
Somatic illness	Yes	64	38.5%
	No	102	61.5%
Distributions of somatic pathologies	Musculoskeletal system	43	67.2%
	Dermatological	23	35.9%
	Gastrointestinal	32	50%
	Coronary	33	51.6%
	Pulmonary	31	48.4%
	Ocular	29	45.3%
	Otolaryngologic	19	29.6%
	Epilepsy	4	6.3%
	Headaches	25	39.1%
	Accidents	20	31.2%
Psychiatric hospitalisation	Yes	43	25.9%
	No	117	70.5%
Psychological therapy	Yes	75	45.2%
	No	91	54.8%
Psychopharmaceutical therapy	Yes	72	43.4%
	No	94	56.6%
Medication	Antidepressants	53	73.6%
	Antipsychotics	19	26.3%
	Anxiolytics	13	18.1%
	Mood stabilisers	9	12.5%
	Hypnotics	6	8.3%
	Psychostimulants	1	1.3%
	Antidementia	0	0%
	Subsidisers	0	0%
	Sexual dysfunction medication	0	0%

Concerning the descriptive data of the therapeutic procedure, it is worth noting (**Table 3**) that on average, clients receive therapeutic aid over the temporal span of 194 days (SD = 249.94) and complete on average 13 (SD = 13.84) psychotherapeutic sessions. Furthermore, 31 individuals terminated their therapeutic process prematurely, which included 18.7% of the whole sample. Thirty-five clients successfully completed psychotherapy, 27 (16.3%) individuals were transferred due to incompatibilities in their therapeutic alliance, and four (2.4%) individuals were not able to consistently receive therapeutic aid. Additionally, 52 (31.3%) individuals had never started the therapeutic process or could not be classified. It must be noted that the deregistration procedure was being reworked and implemented into the data bank used at the clinic during the development of this study. This will allow for a more accurate illustration of this process in future research. The most frequently administered psychotherapeutic modality of the Sigmund Freud Private University Vienna was the systematic approach (n = 33; 19.9%), followed by psychodynamic approaches, like individual or analytic psychotherapy (n = 19; 11.4%). Sixteen clients (9.6%) received cognitive behavioural therapy (CBT), and 14 (8.4%) individuals were treated with humanistic approaches. It is worth noting that cases were acquired by trainee therapists through a procedure that involved clinical judgement by the head of the clinic, the trainee’s supervisor, and their preferences.

**Table 3:** Sociodemographic characteristics of the therapeutic procedure.

Dimensions	Mean	Standard deviation (SD)	
Time in therapy	194.3	249.95	
Psychotherapeutic sessions	13.33	13.84	
Dimensions	Expression	Frequency	Percentage
Therapeutic process	Completed	35	23.5%
	Terminated	31	20.8%
	Transferred	27	18.1%
	Not suited	4	2.8%
	No data/not started	52	34.9%
Therapeutic modality	Systemic	33	19.9%
	Psychodynamic	19	11.4%
	CBT	16	9.6%
	Humanistic	14	8.4%

CBT, cognitive behavioural therapy.

Psychopathological profiles at the Sigmund Freud University Clinic are determined through a multi-step registration process at different points in time and lastly through the clinical judgement of the trainee therapist once the client completes at least five sessions of therapy. While clinical interviews and first interviews are part of this process and a sufficient tool to determine the severity of the clients’ symptoms as well as inform adequate case allocation, the nature of this process does not allow for extensive evidence-based psychological assessments. The diagnoses listed in this study are assessed at the first interview level (third appointment in the registration process) by a licensed psychotherapist.

When analysing these psychopathological profiles of the psychopathological load that clients indicate (**Table 4**), we were able to assess that 22 of the 166 individuals (13.3%) were diagnosed with an F4 classification code after the ICD-10 manual written by the World Health Organization (16). This code encompasses phobic and other anxiety disorders, obsessive–compulsive disorders, reactions to severe stress and adjustment disorders, dissociative disorders and somatoform disorders, and other neurotic disorders. Additionally, 10 clients (6%) were clinically classified with an F3 (affective disorders) diagnostic code. Although only one person (.6%) was diagnosed with an F1 code, which categorises disorders caused by psychotropic substances, 22 individuals (13.3%) of the sample stated that they use alcohol or drugs in a pathological manner. Finally, the remaining persons did not receive a psychiatric diagnosis (n = 133; 80.1%). Sixty-four clients (38.6%) stated life fatigue when initially starting their therapeutic process, and 28 persons (16.9%) were suicidal at the beginning of therapy.

**Table 4:** Sociodemographic characteristics of the psychopathological client load.

Dimensions	Expression	Mean	Standard deviation (SD)
CORE-OM	Total score	1.75	.66
	Well-being deficits	2.2	.89
	Functioning deficits	1.77	.79
	Risk behaviour (self or others)	.37	.48
	Problems/symptoms	2.26	.83
Dimensions	Expression	Frequency	Percentage
ICD-10 diagnosis	F0	2	1.2%
	F1	2	1.2%
	F2	7	4.2%
	F3	56	33.7%
	F4	80	48.2%
	F5	6	3.6%
	F6	9	5.4%
	F7	0	0%
	F8	0	0%
	F9	0	0%
	No mental health diagnosis	4	2.4%
Alcohol or drug abuse	Yes	22	13.3%
	No	144	86.7%
life fatigue	Yes	64	38.6%
	No	102	61.4%
Suicidal	Yes	28	16.9%
	No	138	83.1%

CORE-OM, Clinical Outcomes in Routine Evaluation – Outcome Measure.

**Figure 1:**
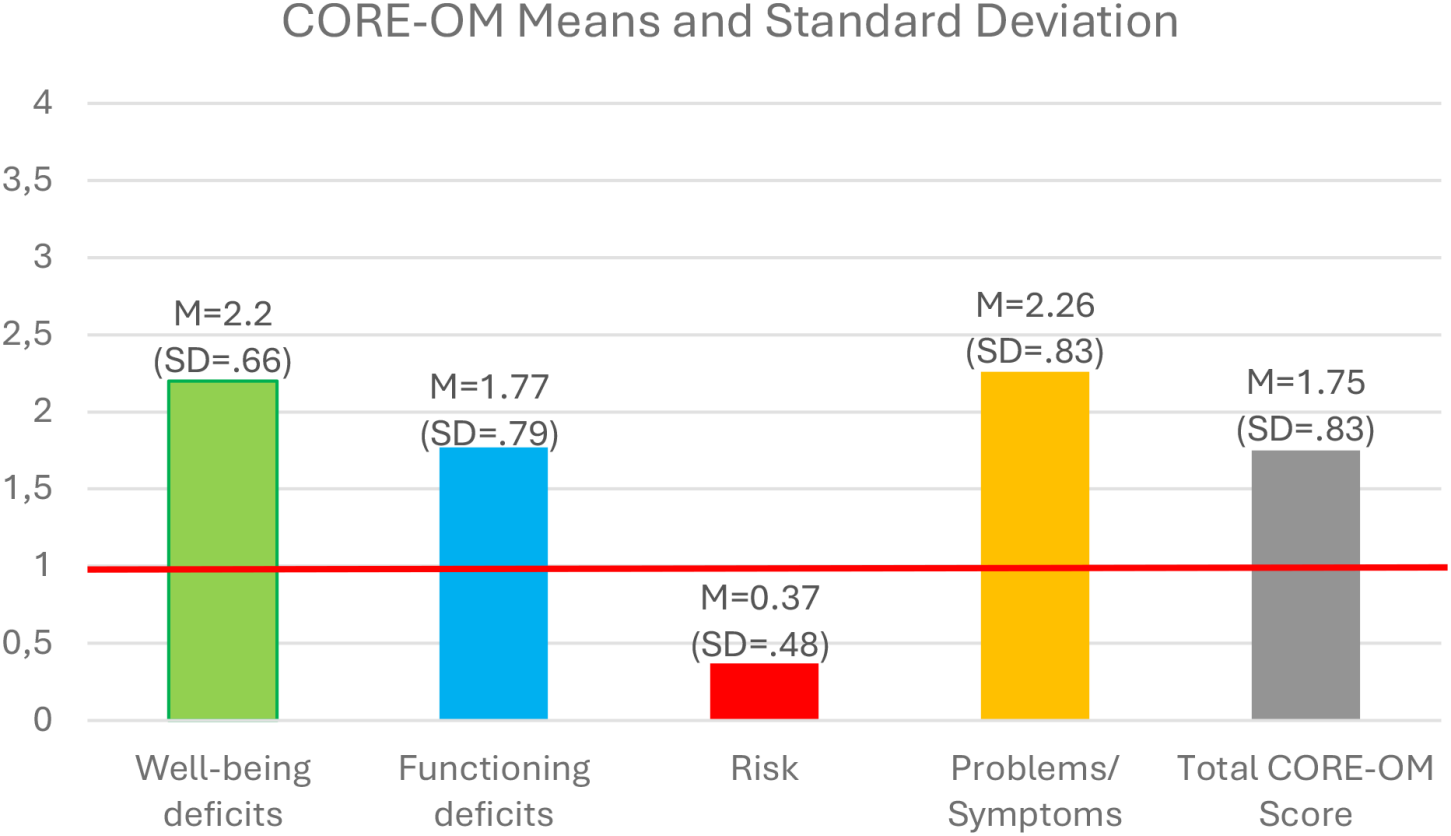
CORE-OM mean scores at the first timepoint and the clinically relevant threshold. CORE-OM, Clinical Outcomes in Routine Evaluation – Outcome Measure.

Apart from these, clients evince the following self-evaluated CORE-OM profile at the beginning of their therapeutic process (T1): on the level of well-being deficits, test subjects showed a mean score of 2.18 (SD = .89); in the domain of risk-taking behaviour (to self or others), an average score of.37 (SD = .48); in functioning deficits, a mean score of 1.77 (SD = .79); and in problems/symptoms, a score of 2.26 (SD = .83). Thus, a total average score of 1.75 (SD = .66) was generated by the sample at the beginning of psychotherapy (**Figure 1**).

### 4.2 Results of the second research question: differences in psychopathological profiles

The analysis of the differences between the three age groups (reflecting different developmental stages) of clients of the psychotherapy outpatient clinic for adults of the SFU concerning the clinical health outcome showed that there is only a significant difference between the three age groups (0 = early, 1 = middle, and 3 = late adulthood) in the subdimension of “well-being” [F(2, 5,042) = 8.18, p <.001, η^2^ <.01] (**Table 5**). The other subdimension and the total CORE-OM score showed no significant differences between the age groups concerning the psychopathological profile (p >.05) of clients partaking therapeutic interventions at the psychotherapy outpatient clinic for adults of the SFU. The statistical parameters of these results can be seen in **Table 5**.

**Table 5:** Results of the one-way ANOVAs between the different developmental stages.

Dimensions	F	df1	df2	P-value	η^2^
CORE-OM: Subjective well-being	8.18	2	5042	<.001	.003
CORE-OM: Total score	.69	2	5042	.5	<.001

CORE-OM, Clinical Outcomes in Routine Evaluation – Outcome Measure.

Due to the lack of variance homogeneity concerning the three subdimensions, “risk”, “functioning”, and “problem/symptoms”, we conducted the alternative Brown–Forsythe test, which is a non-parametric instrument (**Table 6**). These results showed no significant differences between the different age groups concerning the psychological profiles of the geriatric clients (CORE-OM scores) from the outpatient clinic of the SFU (p >.05).

**Table 6:** Results of the Brown–Forsythe test between the different developmental stages.

Dimensions	F	df1	df2	P-value	η^2^
CORE-OM: Problems/symptoms	.17	2	597.77	.84	<.001
CORE-OM: Functioning	.45	2	505.56	.637	<.001
CORE-OM: Risk	1.29	2	888.92	.277	<.001

CORE-OM, Clinical Outcomes in Routine Evaluation – Outcome Measure.

**Figure 2:**
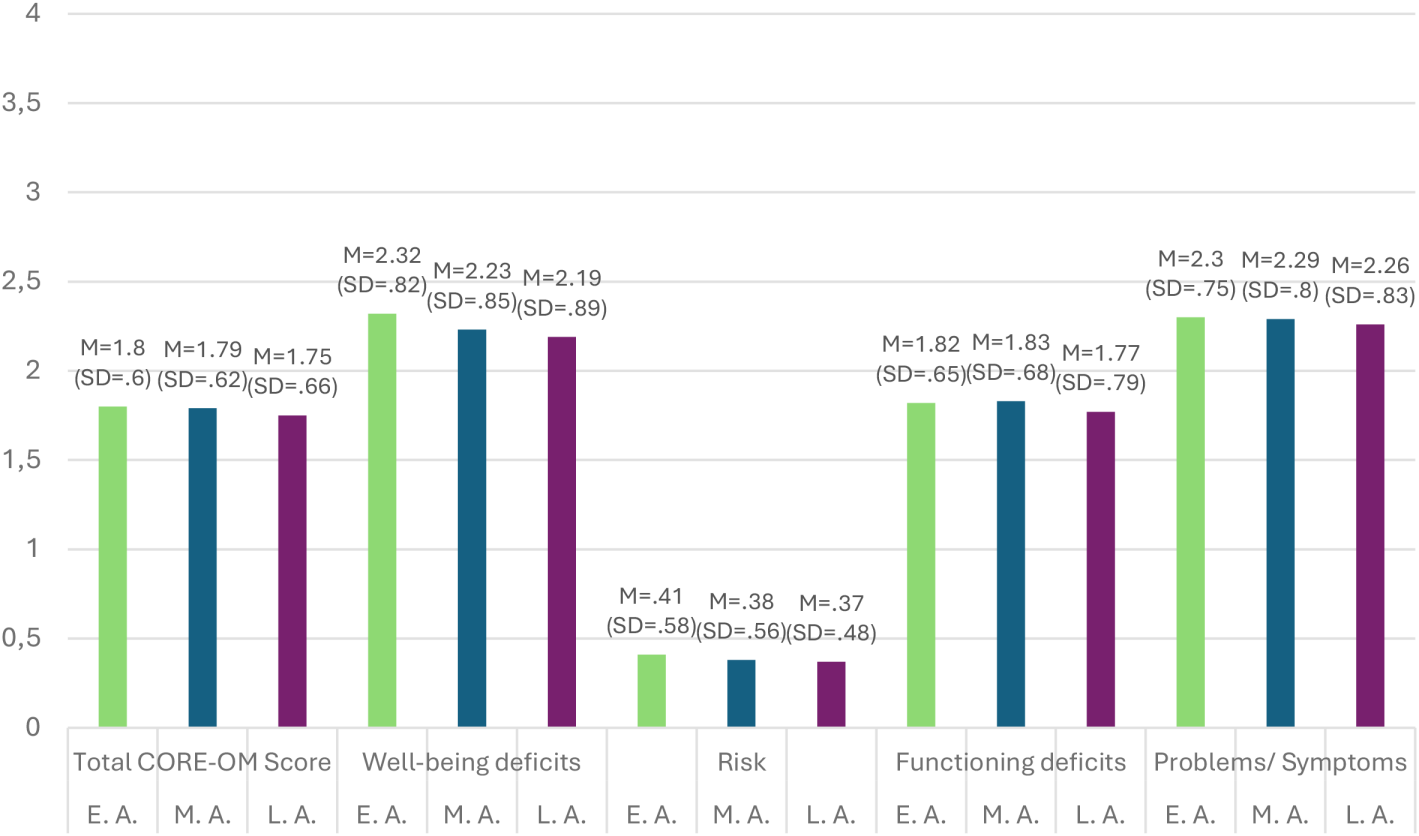
Comparison of the CORE-OM scores based on age group. E.A., early adulthood; M.A., middle adulthood; L.A., late adulthood; Clinical Outcomes in Routine Evaluation – Outcome Measure.

The *post-hoc* analysis between the age groups concerning the subdimension “well-being” showed that individuals who can be classified as young adults differed significantly in their well-being deficits from clients who entered the developmental stage of middle adulthood (p <.001). There were no significant differences between the psychopathological well-being profile of older adult individuals in contrast to the other developmental stages or the remaining groups (p >.05); for all descriptive statistics see **Figure 2**.

The results of the paired χ^2^ analyses indicate (**Table 7**) that the defined age groups (early, middle, and late adulthood) were significantly associated with the therapeutic process, showing higher tendencies from individuals in middle adulthood (z = 2.7; 24.7%) in premature therapeutic termination behaviour as well as lower expression rates in transferring therapy (z = −3.6; 20.5%) than from the frequency peculiarities expected. Older adult adults showed high therapeutic termination rates (20.8%), but this effect was not significantly expressed compared to that in the other age groups. The remaining results suggest that older adult adults can be significantly associated with educational level [suggesting higher occupational tendency (z = 3.9; 19.3%) in lower educational levels for geriatric clients], existence of somatic illnesses [showing higher illness expression for older adult individuals than from the frequency peculiarities (z = 2.4; 51.2%) expected], prior psychopharmaceutical treatments [showing higher occupational values (z = 3.9; 43.3%) in consuming medication than expected for gerontopsychological patients], and alcohol or drug abuse behaviour [indicating lower abuse (z = 3.6; 86.7%) than expected from persons in late adulthood]. Life fatigue, suicidality, and prior psychotherapy do not seem to significantly correlate with older adult. This effect cannot be perceived overall, illustrating that only life fatigue is not associated with the age groups (p >.05).

**Table 7:** Results of the paired χ^2^ analyses between the different age groups.

Dimensions	χ^2^	df	c	c_corr_	P-value
Therapeutic process	45.79	8	.1	.12	<.001
Educational level	305.04	10	.24	.28	<.001
Somatic illness	24.21	2	.07	.09	<.001
Psychological therapy	30.66	2	.08	.1	<.001
Psychopharmaceutical therapy	171.19	2	.18	.24	<.001
Alcohol or drug abuse	195.08	2	.19	.26	<.001
Weariness of life	1.13	2	.02	.03	.568
Suicidal	11.26	2	.05	.07	.004

## 5 Discussion

The results of this analysis provide valuable comprehensive insights into the psychosocial profiles and characteristics of psychotherapeutic treatment for older clients in an outpatient psychotherapeutic setting. 

The discussion is structured according to our research questions: RQ I (specific sociodemographic characteristics and (bio-)psychosocial stressors of older clients) and RQ II (differences from younger age groups) are addressed in sections 5.1, 5.2, 5.3, and 5.4. Section 5.5 derives implications for care and training.

### 5.1 Mental health and physical illness in older adult populations

RQ I: The present findings on somatic comorbidities and their functional consequences characterize the (bio-)psychosocial profile of older adult clients in this setting. RQ II: Comparison with younger groups and with external reference data suggests a potential underreporting of somatic illnesses among older adult clients in this sample.

RQ I: Relational effects between mental health and physical health have been investigated in multiple studies including a recent meta-analysis (17), overwhelmingly concluding that patients with severe mental illnesses are at higher risk of suffering from physical comorbidities than the general population and having decreased life expectancies (18–25). Furthermore, general healthcare needs are commonly neglected in patients with severe mental health issues (26); some research suggests that the deterioration of physical health can contribute to further clinically relevant mental health episodes (27), while others tend to point out the bidirectionality of mental and physical health factors (28).

Although all contributions mentioned above emphasise the importance of considering physical health factors when treating patients with mental health disorders, the relationship between the two remains both complex and inseparable. This fact is probably most prominently established by the cross-government white paper “No Health Without Mental Health” (29).

RQ II: Although there is no population record to our knowledge in Austria that aligns with the dimensions of somatic illnesses as defined by the clinic’s registration procedure, Statistik Austria’s 2019 health report investigated the effect of physical illness on the day-to-day life of the Austrian population. This investigation concluded that 48.7% of people over 60 suffer from physical constraint in their daily activities (for at least the past 0.5 years) due to physical illnesses, compared to only 22.1% of people between ages 15 and 65 (30). In contrast, the study findings only showed that 38.6% of people questioned reported suffering from somatic illnesses. This is worth noting, especially since the presence of a somatic illness alone does not entail limitations in day-to-day activities, but there must be a somatic illness present for it to be a limiting factor. Therefore, one would expect the percentage of reported illnesses in the sample to be even higher than the one reported by Statistik Austria. Furthermore, as discussed above, physical illnesses can be seen as both a cause and an effect of mental health problems, which would imply an even higher percentage of physical illnesses in the study sample than in the general population.

The cause of this discrepancy remains unclear though one possible explanation could involve clients intentionally withholding information on their physical health either because they do not expect any help regarding these issues from a mental health institution or they feel that the presence of physical illnesses is not of concern in mental health treatment. There are several other possible explanations which would involve even higher degrees of speculation. For now, it can be concluded that a higher number of underreported cases of physical illnesses in this client population are expected. This is especially troublesome in light of the complex connection between mental and physical health discussed above as well as the increased presence of constraints due to physical illness in older populations (30). Self-reported somatic conditions may be underreported in this cohort. Older adults sometimes normalise chronic physical complaints or prioritise psychological concerns during intake, which can lead to underdocumentation of physical comorbidities. In addition, it is possible that the older clients who actively seek outpatient psychotherapy represent a comparatively physically fitter subgroup within this sociodemographic context, while individuals with higher somatic burden may either not access psychotherapy or remain underrepresented in our sample. Future studies should therefore include brief qualitative follow-up to better validate and contextualise the physical health burden in older psychotherapy clients. In response to the possible underreporting of somatic illnesses, a brief qualitative follow-up examination could be integrated into the admission process. This could include (i) a more detailed medical history in the event of somatic problems, supported by short topic-specific guidelines and questionnaires to deepen the assessment and provide information about subsequent treatment, as well as a clear checklist of common diseases and functional effects derived from the current literature to ensure more comprehensive coverage. Based on this, the clinic is already developing its own structured supplementary medical history tool in the form of a guided interview with an integrated body-mind focus to expand the assessment across all cases.

These conclusions may also apply to other mental health institutions and therefore emphasise an increased need for mental health professionals to monitor physical issues more closely and recommend referring clients to specialists if necessary.

### 5.2 Psychopharmaceuticals and age

RQ II: Another result in this analysis involves the relatively high percentage (43.4%) of individuals in our sample already prescribed at least one psychopharmaceutical. An Austrian study from 2022 showed that 12.4% of the total population were prescribed at least one psychopharmaceutical in 2020 (31). However, this study did not specifically refer to the 60+ age group. In contrast, the prevalence in retirement and nursing homes is much higher (32) and exceeds the observation of this study (**Table 2**). This may be due to the readily available medical support in retirement homes and the relative ease with which medication can be accessed through it, compared to a population visiting an outpatient clinic. This type of treatment presents another challenge for mental health professionals, as psychopharmaceuticals tend to have various adverse effects on older adult clients, but are not present in younger cohorts. These include pharmacokinetic changes like differences in absorption, metabolism, excretion, and distribution, as well as pharmacodynamic changes like sensitivity increases and decreases of certain neuronal receptors (33). Although these age-related changes in the body are well documented and researched, few systematic trials are conducted in older populations to determine optimal dosages (34). Therefore, an increased awareness by psychotherapists towards unusual side effects even with generally well-tolerated medication may be warranted, especially considering that roughly half of the sample evaluated may be affected by this issue. These results underscore the need for age-adapted pharmacological considerations in the interpretation of outcomes and side effects regarding the RQ II and briefly explain the relevance of medication-related (bio-)psychosocial stressors in the profile of older clients regarding the RQ I.

### 5.3 Therapy retention and premature termination rates in older adult clients

This section provides path-relevant therapy characteristics in the profile of older clients in regard to RQ I. The average duration of ongoing psychotherapeutic treatment for older individuals is approximately 14 sessions. Premature termination of therapy remains an essential challenge in this demographic. Research into dropout rates in psychotherapy suggests various contributing factors, including the quality of the therapeutic alliance, perceived effectiveness, and the expectations unique to this client group. Whether patients independently choose to terminate therapy plays a decisive role (35).

Reasons for early discontinuation often involve mismatches between therapist and patient, highlighting the critical importance of the therapeutic relationship and the alignment of expectations tailored to the older population. Addressing these factors during initial treatment allocation, such as through personalised matching processes, can significantly reduce the risk of dropouts. Thus, optimising intake assessments and screening processes, emphasising comprehensive biographical exploration and prior therapeutic experiences, can help establish stronger therapeutic alliances. These alliances are essential for achieving positive outcomes, particularly within geriatric populations (36).

### 5.4 Psychopathological profiles and well-being

RQ I: The analyses and CORE-OM results describe the psychopathological profile of older clients (including higher somatic burden, lower substance abuse rates, lower well-being). RQ II: Age group differences in well-being are clear, while the severity of life fatigue does not systematically correlate with the stage of development.

RQ I: The chi-square analyses revealed significant associations between developmental stages and other factors such as educational level, somatic diseases, previous treatments, and suicidality. Geriatric clients had a higher prevalence of somatic diseases, which is congruent with existing literature in geriatrics (37). In contrast, the lower incidence of substance misuse in late adulthood compared to younger age groups represents a contrast.

The finding that developmental stages do not correlate significantly with aspects of life fatigue raises suspicious questions about the generalisability of existential concerns in older adulthood. This implies that the aspect of life fatigue is a general problem of adulthood. Future research could investigate whether certain life events or psychological states contribute to this phenomenon, potentially offering new therapeutic interventions.

RQ II: While these effects provide interesting insights into overlaps and contrasts between different age cohorts, it should be noted that the dimensions assessed by the CORE-OM (functionality, well-being, risk, and problem) can have different effects depending on this special age range. For example, lower scores in functioning and the associated impact on occupational status approaching retirement age may be interpreted differently. Although these factors are complex and interdependent, they reflect previous research on the applicability of the CORE-OM in older adult clients (38). Applied to the clinical setting, this would mean that a pattern of lower well-being with largely similar problem/risk scores would suggest that adaptation for older adults should focus less on acute risk management and more on improving meaningfulness, social connectedness, autonomy, and coping with multimorbidity. This suggests that interventions for older adults should place greater emphasis on loneliness, themes of loss and bereavement, the preservation of autonomy, and the integration of somatic burdens into treatment.

### 5.5 Implications for Clinical Practice and Training

The findings derived from RQ I and RQ II support age- and context-sensitive care models (e.g., more accessible facilities, integrated treatment of somatic and psychological issues).

This study highlights the importance of tailoring psychotherapeutic services to the specific psychosocial and clinical needs of older clients. Beyond clinical practice, the study aims to make recommendations for improving these services. Health policy should promote age-appropriate, country-specific mental healthcare structures, improve access to affordable psychotherapeutic treatments for older adults and enable group therapies and self-help groups in nearby or central locations. Group formats such as group therapy and self-help groups are particularly valuable, as they also reduce loneliness and strengthen social bonds. Meta-analyses of group activities such as memory work/life review and community activities show significant benefits for well-being (39). This approach should also be expanded and strengthened. Additionally, psychology and psychotherapy training programs should adopt a clinical gerontopsychological focus, both in theory and in practice, to prepare future professionals for the growing demand in this area. For example, undertaking an internship during the first phase of study in nursing homes and long-term care facilities with a psychosocial focus. Taking these clinical, political and educational factors into account can make psychotherapeutic care for older adults more responsive to needs and effective.

Moreover, RQ I shows that older clients in this setting are characterized by specific (bio-)psychosocial profiles that are directly relevant for diagnosis, treatment planning, and allocation. RQ II also demonstrates differences compared to younger, while some existential dimensions are not consistently age dependent. This leads to concrete clinical, organizational, and training-related measures that improve the suitability of interventions in older age.

## 6 Limitations

### 6.1 Sample size and generalisability

A sample size calculation was not feasible because the study draws on retrospectively available intake records from an outpatient setting. While the sample (n=166) provides solid descriptive insight into older clients in outpatient psychotherapy, generalizability beyond this clinic, especially to services dedicated to gerontopsychological care or to other regions, may be limited. The authors encourage readers to interpret subgroup findings with appropriate caution given limited power; the results are best viewed as a transparent basis for future, confirmatory studies with larger samples.

### 6.2 Cultural and socioeconomic factors

The study takes place in a specific cultural and socioeconomic context, in a psychotherapeutic outpatient clinic in Vienna. Cultural factors such as the stigmatization of mental health or different attitudes towards ageing may influence the way older people perceive psychotherapy and are willing to engage in it. In addition, socioeconomic variables such as access to healthcare and financial resources were not controlled for in detail, which could influence the results. 

In Austria, access to outpatient psychotherapy is partly shaped by a mixed reimbursement system, in which publicly subsidized or insurance-covered therapy slots are limited and often require waiting time, while fully self-funded treatment remains a financial barrier for many clients. These structural and cultural factors must be considered when interpreting the generalizability of our findings beyond the Viennese/Austrian context.

### 6.3 Discontinuation rates and diagnoses

The diagnoses of the sample in the psychotherapeutic outpatient clinic are made in a very low-threshold registration process by clinically trained staff (licensed psychotherapists) and at the start of therapy and are descriptive. These intake diagnoses reflect preliminary clinical impressions rather than longitudinal or fully validated diagnostic workups. This should be considered when interpreting diagnostic categories in the results, as some diagnostic misclassification cannot be excluded. This can lead to diagnostic inaccuracy. At this stage, the focus is placed on essential psychotherapeutic aspects relevant to treatment; a comprehensive psychological diagnosis is only recommended if indicated. This may be a significant factor contributing to the high proportion of ICD-10 F3 and F4 diagnoses. Therefore, we included CORE-OM scores and other markers (suicidality, alcohol/drug-abuse, etc.) to meaningfully characterize our clients’ psychological stress/strain profile.

#### 6.3.1 Allocation to therapeutic modality

Therapist were assigned based on assessment of the clinic team, trainee competencies, preference of the patient, clinical fit, and practical availability, to guarantee optimal and as-rapid-as-possible care. This procedure is clinically appropriate in a real-world outpatient setting but introduces selection bias.

## 7 Conclusion

Finally, the analysis showed that there is a great need not only to research this special age group in more detail but also to take up the corresponding resources preventively to sustainably increase the quality of life over the life span. It is essential to create awareness for this questionable age group already in the training of the various health professions so that interest in it can bear fruit at all. The complexity between physical and psychological problems is intertwined and cannot be clearly separated in the treatments, so these clients can also be cared for in the long term. In addition, extensive (psycho)pharmacological training in the gerontopsychological field would be useful so that psychotherapists and practitioners at all times can recognise possible problems on an interaction basis or learn to understand the connection to be able to recommend appropriate clarifications. The health professions need an all-encompassing knowledge of this age group concerning initiating adequate interventions and treating comorbidities, as well as meeting the expectations of this age group or even clarifying adequate and suitable offers of therapy. The data and experience of the outpatient clinic should help to enhance this awareness and knowledge.

The question about implications for outpatient care and future directions describes in detail how tailor-made therapeutic approaches for older people can be developed. Based on the results, future research should aim to develop and refine therapeutic interventions that are specifically tailored to the needs of older adults and consider the appropriate profiles. An important aspect is digitisation in medical and gerontopsychological care; this could explore future research on the feasibility and effectiveness of technology-enabled interventions for older clients. Online psychotherapy, applications, and virtual reality could be explored as potential tools to provide more accessible and flexible treatment options, especially for older adults with mobility or transportation issues. The new generations are already familiar with technology, so it may even be impossible to imagine life without it in old age. Studies investigating the effectiveness of digital treatments alongside traditional face-to-face therapy could not only expand but also revolutionise the range of therapeutic resources available to this population.

## Data Availability

The datasets for this article are not publicly available due to ethical and legal restrictions related to the protection of participant confidentiality and privacy, as the data include sensitive information from an older psychiatric population. Requests to access the datasets should be directed to the corresponding author.
